# Supplementary material for: Wastewater Surveillance of SARS-CoV-2: A Comparison of Two Concentration Methods
Source: Viruses. 2024 Aug 31;16(9):1398. doi: 10.3390/v16091398 (PMC11436116; doi:10.3390/v16091398)
Supplement: Supplementary file 1 [file viruses-16-01398-s001.zip › viruses-3168218-supplementary.pdf]

Table S.1: Summary table with the Ct measurements of all samples.

SAMPLE	Ct Numbers for SMF	Ct Numbers for PEG
1	36,90	36,76
2	-	37,51
3	38,71	37,84
4	-	38,23
5	39,44	37,33
6	-	37,74
7	-	35,24
8	-	36,91
9	-	-
10	-	40,03
11	-	38,74
12	38,91	38,30
13	-	35,31
14	-	-
15	-	35,87
16	-	36,70
17	-	36,10
18	41,14	36,51
19	-	36,39
20	-	35,49
21	-	39,69
22	40,18	37,50
23	-	37,62
24	38,03	35,89
25	39,81	38,99
26	-	40,67
27	-	37,36
28	36,99	36,21
29	38,64	35,60
30	38,70	36,04
31	38,69	33,91
32	-	33,15
33	42,59	34,14
34	35,15	33,20
35	34,29	33,09
36	35,18	36,32
37	34,22	34,62
38	37,22	35,05
39	37,68	39,08
40	38,55	37,22

Table displaying the Ct numbers of each sample.

Table S.2: Positivity and negativity of samples using two different Ct thresholds.

Threshold 40		
SMF - PEG	+	-
+	17	0
-	19	4

Threshold 45		
SMF - PEG	+	-
+	20	0
-	18	2

Table S.3: Quantification results of Skimmed Milk Flocculation method and PEG precipitation.

SAMPLE	DATE	QUANTIFICATION SMF (GC/L)	QUANTIFICATION PEG (GC/L)
1	2/6/2021	1,33E+04	1,46E+04
2	7/6/2021	0,00E+00	8,79E+03
3	8/6/2021	3,91E+03	7,04E+03
4	14/6/2021	0,00E+00	5,41E+03
5	15/6/2021	2,40E+03	9,94E+03
6	16/6/2021	0,00E+00	7,53E+03
7	21/6/2021	0,00E+00	4,06E+04
8	22/6/2021	0,00E+00	1,32E+04
9	23/6/2021	0,00E+00	0,00E+00
10	28/6/2021	0,00E+00	1,61E+03
11	29/6/2021	0,00E+00	3,84E+03
12	30/6/2021	3,43E+03	5,15E+03
13	5/7/2021	0,00E+00	3,88E+04
14	6/7/2021	0,00E+00	0,00E+00
15	7/7/2021	0,00E+00	2,66E+04
16	13/9/2021	0,00E+00	1,52E+04
17	15/9/2021	0,00E+00	2,28E+04
18	16/9/2021	7,63E+02	1,73E+04
19	22/9/2021	0,00E+00	1,87E+04
20	23/9/2021	0,00E+00	3,43E+04
21	27/9/2021	0,00E+00	2,03E+03
22	28/9/2021	1,45E+03	8,85E+03
23	4/10/2021	0,00E+00	8,16E+03
24	5/10/2021	5,74E+03	2,62E+04
25	11/10/2021	2,80E+02	3,24E+03
26	12/10/2021	0,00E+00	1,04E+03
27	19/10/2021	0,00E+00	9,73E+03
28	26/10/2021	1,25E+04	2,11E+04
29	1/11/2021	4,10E+03	3,19E+04
30	3/11/2021	3,94E+03	2,37E+04
31	8/11/2021	3,96E+03	9,97E+04
32	15/11/2021	0,00E+00	1,66E+05
33	16/11/2021	4,88E+02	8,54E+04
34	22/11/2021	4,32E+04	1,61E+05

**35**	23/11/2021	**7,71E+04**	**1,73E+05**
**36**	29/11/2021	**4,23E+04**	**1,96E+04**
**37**	30/11/2021	**8,09E+04**	**5,58E+04**
**38**	6/12/2021	**1,07E+04**	**4,62E+04**
**39**	13/12/2021	**8,40E+03**	**3,05E+03**
**40**	14/12/2021	**4,36E+03**	**1,07E+04**

Table of results displaying the dates of sample receipt and quantification of results expressed in Genome copies/L.

Table S.4: Correlation of physicochemical parameters.

**PEARSON CORRELATION**						
	**pH**	**EC**	**COD**	**BOD**	**Ptot**	**NH4N**
**Coefficient (r)**	-019	0,02	-0,07	-0,20	-0,03	0,13
**p value**	0,12	0,56	0,33	0,10	0,42	0,78
